# Internal bone transport using a cannulated screw as a mounting device in the treatment of a post-infective ulnar defect

**DOI:** 10.1007/s11751-016-0246-6

**Published:** 2016-02-15

**Authors:** Konstantinos Tsitskaris, Heledd Havard, Paulien Bijlsma, Robert A. Hill

**Affiliations:** Department of Orthopaedics, Great Ormond Street Hospital for Children, London, WC1N 3JH UK; Department of Orthopaedics, St Mary’s Hospital and Hillingdon Hospital, London, UK

**Keywords:** Internal bone transport, Ulnar segmental bone defect, Cannulated screw, PVL *S. aureus*

## Abstract

Bone transport techniques can be used to address the segmental bone loss occurring after debridement for infection. Secure fixation of the bone transport construct to the bone transport segment can be challenging, particularly if the bone is small and osteopenic. We report a case of a segmental ulnar bone defect in a young child treated with internal bone transport using a cannulated screw as the mounting device. We found this technique particularly useful in the treatment of bone loss secondary to infection, where previous treatment and prolonged immobilisation had led to osteopenia. This technique has not been previously reported.

Segmental bone loss can occur as a consequence of fractures, after tumour resection and following extensive debridement for bone infection [1]. Treatment methods used to address large bone defects include traditional bone grafting, vascularised bone grafts, the induced membrane technique and bone transport techniques [2]. Secure fixation of the bone transport construct to the bone transport segment can be challenging, particularly if the bone is small and osteopenic. We report a case of a segmental ulnar bone defect in a young child treated with internal bone transport using a cannulated screw as the mounting device. This technique has not been previously reported.

## Case report

A 2-year-old girl presented to her local hospital with pain and swelling in the left forearm and elevated inflammatory markers. Plain radiographs and a magnetic resonance imaging scan confirmed the clinical diagnosis of osteomyelitis of the left ulna. She was started on broad-spectrum antibiotics and transferred to our unit where she underwent surgical debridement. Tissue samples were positive for infection with Panton–Valentine leukocidin (PVL) *Staphylococcus aureus* (*S. aureus*), sensitive to flucloxacillin and clindamycin. She underwent two further surgical debridements and received a six-week course of intravenous antibiotics. The inflammatory markers returned to normal levels, and the debridement wounds healed with no local sequelae.

**Fig. 1:**
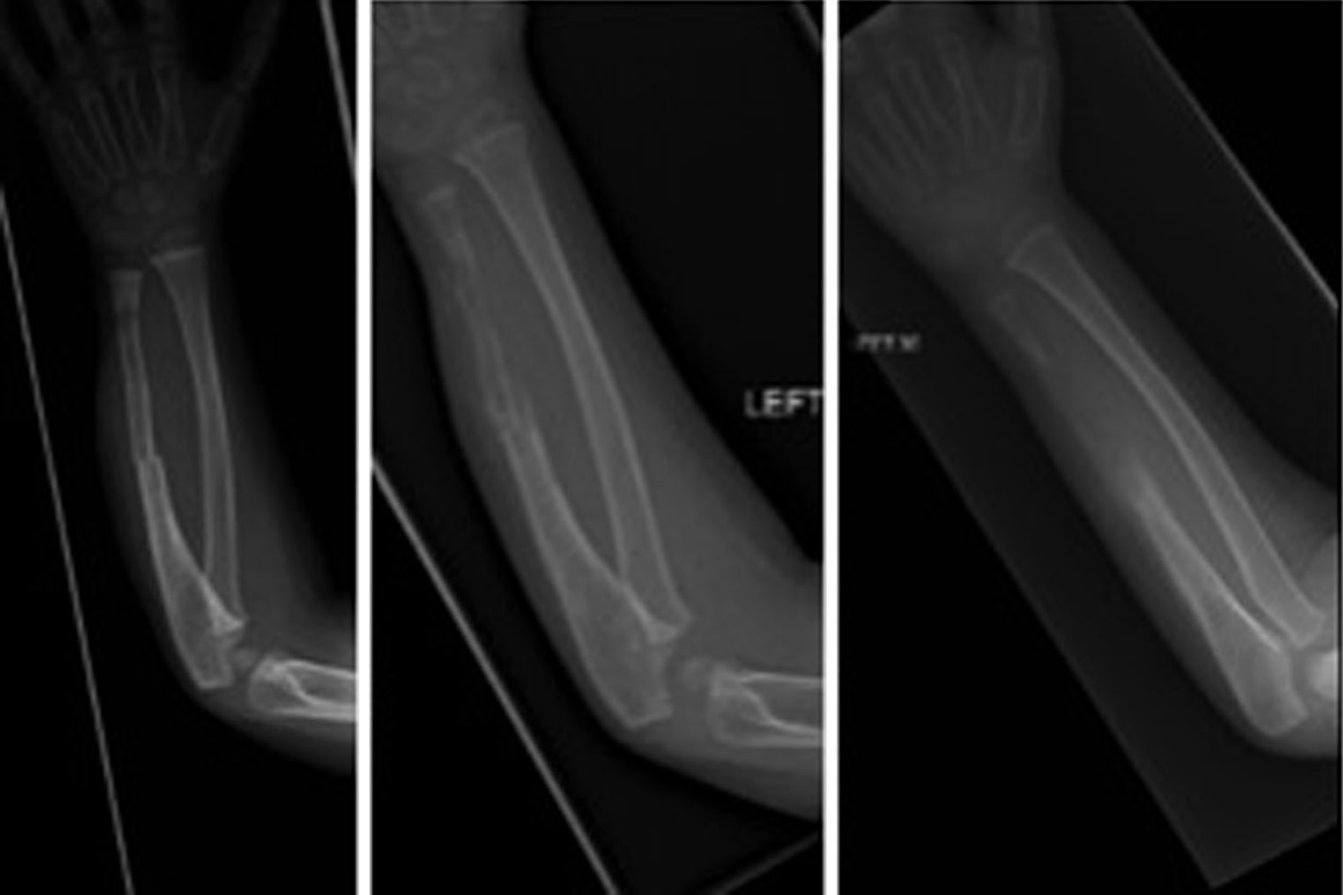
Significant osteolytic process affected the left ulna with dissolution of part of the bone

During the following 3 months, a segment of the ulna became osteolytic and after minor trauma, a midshaft fracture occurred which was treated in a cast (Fig. 1). Substantial dissolution of the ulnar shaft took place, leaving the distal ulna and its growth plate radiographically intact. During this period, the patient was assessed at frequent intervals and no clinical or biochemical evidence of an active infection was identified. The ulnar bone defect measured approximately 3.5 cm and showed no evidence of recovery. After the osteolytic process had reached a plateau on sequential radiographs, ulnar reconstruction was undertaken.

### Surgical technique

**Fig. 2:**
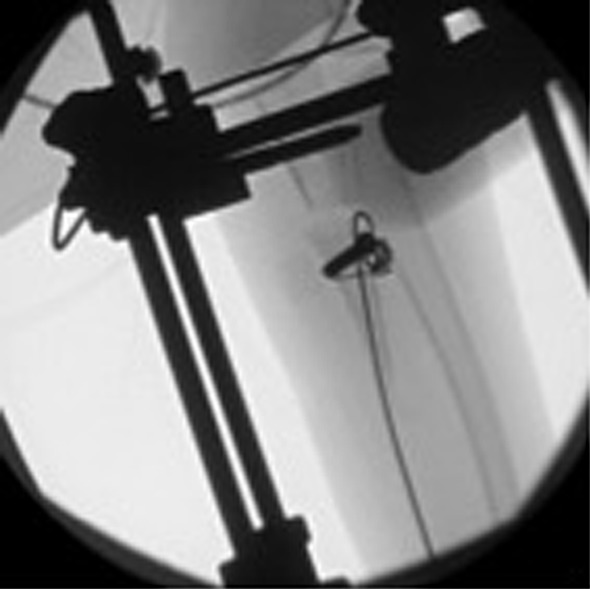
Bone transport segment is secured on a traction wire by threading the latter through a 3.5-mm cannulated screw positioned perpendicular to the long axis of the bone

**Fig. 3:**
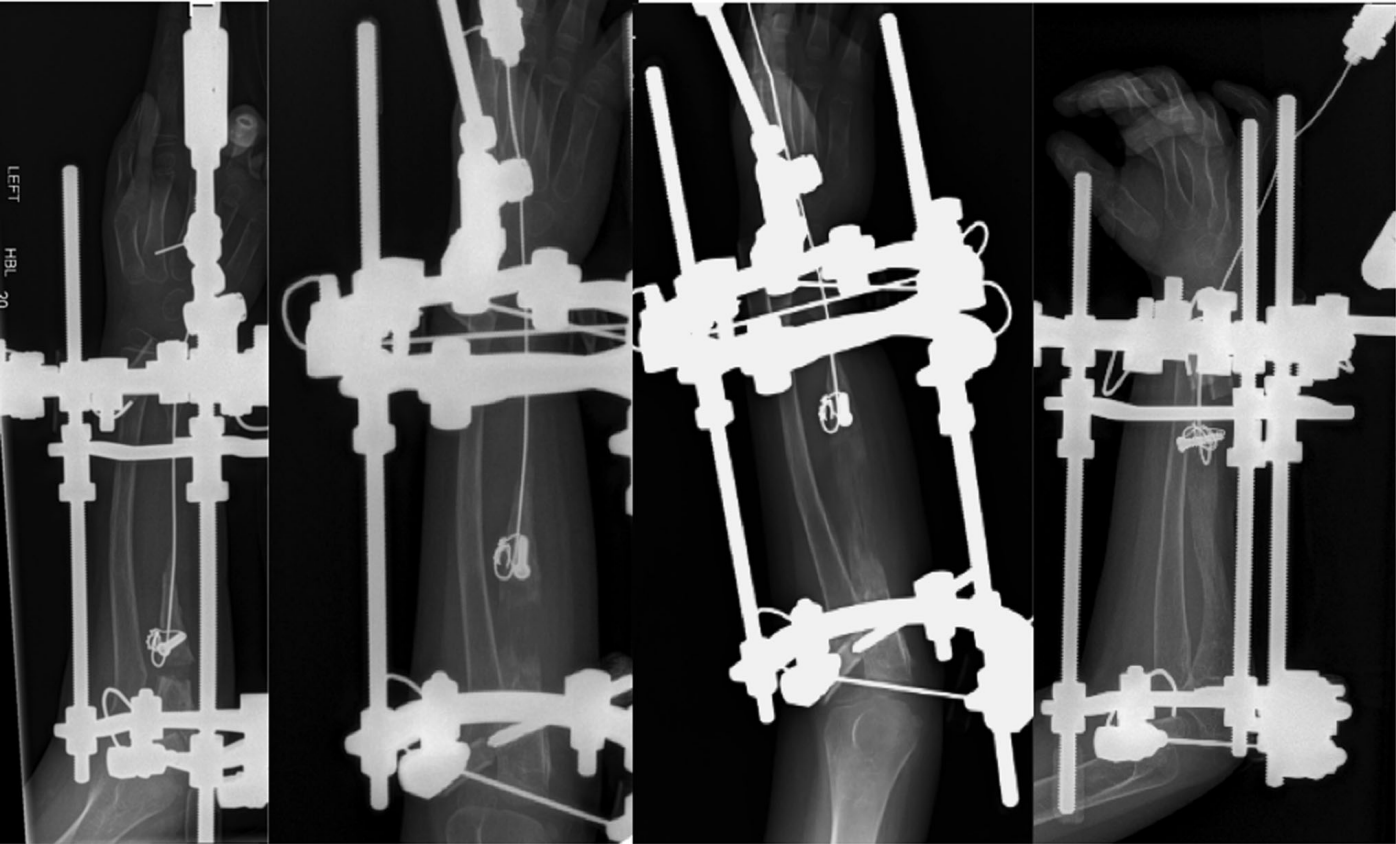
Ulnar defect was bridged and consolidated during the course of the treatment

An Ilizarov fixator (Smith & Nephew Orthopaedics Ltd, Warwick, UK) was used. The ulnar osteotomy was performed percutaneously by pre-drilling the bone prior to attaching the frame. The level for the osteotomy was the proximal metaphysis just distal to the coronoid process. Initially, the Ilizarov fixator was set up for external transport, with a proximal half ring, a half ring for bone transport and a distal complete ring using the elevator method. The osteotomy was then completed with an osteotome after attachment of the frame. Within the first 2 weeks of treatment, the transport wire pulled through the osteopenic bone, necessitating revision. At the time of revision, the treatment was converted to an internal bone transport construct. We secured the bone transport segment with a 1.5-mm transport wire by threading the wire through a 3.5-mm cannulated screw, positioned perpendicular to the long axis of the bone (Fig. 2). The transport wire exited at the level of the wrist and was mounted to a slotted rod distraction device (Fig. 3).

**Fig. 4:**
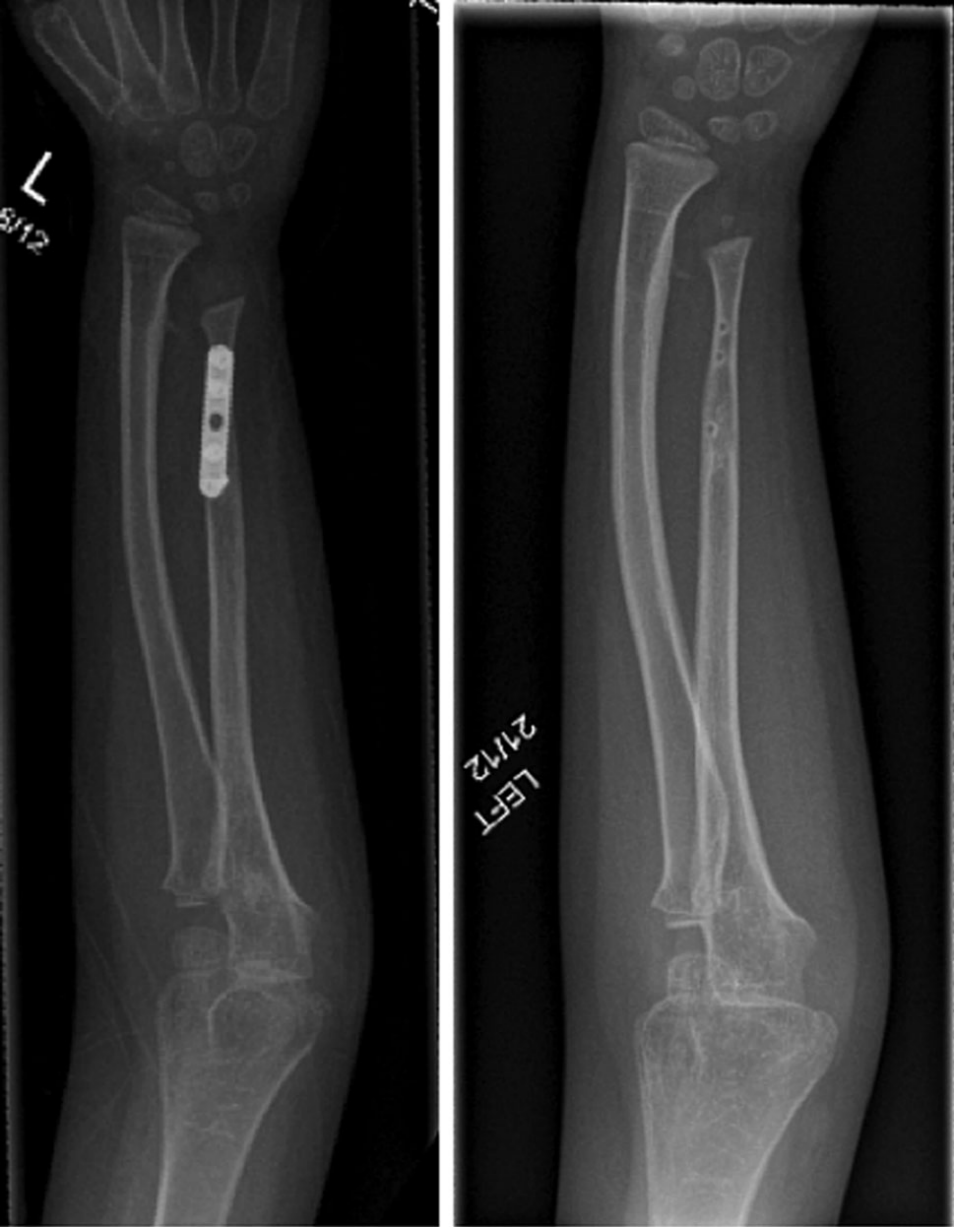
Fixation of the docking site (*left*) and the end result (*right*)

The distraction started 5 days post-operatively at a rate of 0.75 mm/day. There were no complications in the immediate post-operative period. During the final stages of the bone transport, the patient received a course of oral antibiotics for a pin site infection. During the bone transport, radiographs were taken every 2 weeks in order to monitor new bone formation. After 3 months, the ulnar defect was effectively bridged and consolidated during the following 2 months (Fig. 3). The frame was removed when corticalisation of three out of four cortices was evident on radiographs. During the procedure to remove the frame, the docking site was assessed and stabilised using a small fragment plate; eventually, this was also removed 11 months later (Fig. 4).

The patient had supervised elbow and hand therapy throughout the treatment to maintain function. Initially, passive range-of-movement exercises were undertaken, and eventually, as the pain decreased, active exercises were encouraged. At the final follow-up, 30 months after the initial presentation, the patient remained pain-free and was able to undertake her usual daily activities without restrictions. She had equal range of movement to the contralateral arm, with mildly increased ulnar deviation at the left wrist; the latter was completely pain-free.

## Discussion

Bone transport can be internal or external; in the former, the transport segment is pulled by a construct that is inside the limb exiting distally, whereas during the latter, the bone segment is attached to a transport ring, with the construct moving through the soft tissues. The main advantage of the internal technique is that it eliminates the risk of transection of important structures, such as nerves. The main difficulty is the secure fixation of the internal transport construct to the bone transport segment, particularly if this is small and osteopenic.

**Fig. 5:**
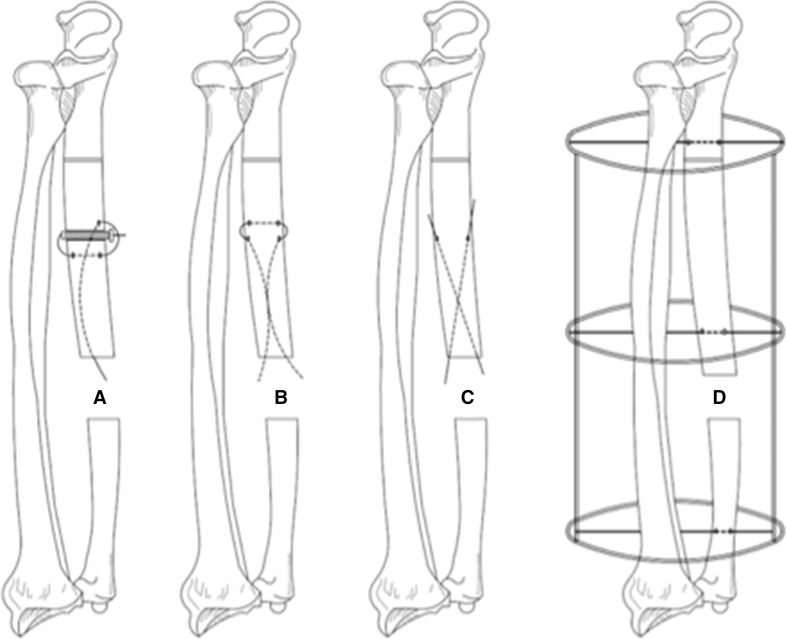
Forearm model for bone transport [**a**, **b** and **c**, internal bone transport (**a** the cannulated screw technique, **b** the flexible cable technique, **c** the olive wire technique). **d** external bone transport]

Commonly used methods for the fixation of the internal bone transport segment include olive wires or flexible cables; both run the risk of “cheese wiring” through the bone transport segment. The use of a cannulated screw–wire construct proved effective in providing a secure mounting point in this case where the transport segment was osteoporotic and an external transport wire had cut out. Figure 5 depicts the cannulated screw technique, the two most commonly used techniques to secure the bone segment during internal bone transport and the external bone transport technique.

Potential alternative options for the treatment of a segmental ulnar bone loss, as in this case, include a vascularised bone graft or the creation of a one-bone forearm [3, 4]. The latter can be accomplished by medial translocation of the radius to place it in continuity with the ulna. It can be associated with an increased incidence of complications and should probably be considered as a last resort option [5]. Vascularised bone grafts usually consist of a fibular segment, but radial and humeral grafts can also be used. The bone is vascularised, remains viable and also has structural strength. The disadvantages of this technique are substantial and include significant complexity, risk of failure of the vascular anastomosis, risk of non-union to the host bone and morbidity of the donor site. A comparison of this method with bone transport in the femur revealed superior results with the latter method [6].

PVL is an exoprotein with increased toxicity to immune cells. It was first described in 1932 and is secreted in approximately 2 % of *S. aureus* infections [7, 8]. PVL-secreting *S. aureus* infections are more aggressive, usually affect the soft tissues and can be complicated by secondary infections such as necrotising haemorrhagic pneumonia [9]. PVL-secreting *S. aureus* osteomyelitis in children is a rare entity with a limited number of reports in the literature [10, 11]. Our patient developed a localised infection with a significant concomitant osteolysis and an almost complete dissolution of part of the ulnar diaphysis. We believe that this was a result of the significant virulence of the pathogen, which is in agreement with previous reports [10].

After a lengthy treatment, our patient enjoys a very functional, painless and infection-free arm. Nevertheless, there is still a residual relative discrepancy in the length of the radius and ulna (Fig. 4). We believe that this is a result of the proximal migration of the distal ulnar, whilst it was not in continuity with the rest of the shaft. In addition, although the osteomyelitic process appeared to be strictly diaphyseal, it may have had an indirect effect on the distal ulnar physis affecting its growth potential.

Patients with a discrepancy in the length of the radius and ulna are at risk of developing symptoms from the adjacent joints. In particular, a shorter ulna predisposes the patient to the risk of subluxation or dislocation of the radial head [12]. Such a deformity has not occurred in this case, and the patient has remained asymptomatic, precluding the need for further treatment. Nevertheless, the patient will continue to be followed up until skeletal maturity. Options for future treatment will depend on whether the discrepancy is progressive or stable and include radial epiphysiodesis with or without further ulnar lengthening, potentially employing the same treatment principles. Such intervention could accomplish a congruent distal radio-ulnar articulation, but would be prone to the risks associated with major limb reconstruction surgery.

## Conclusions

The technique we present here offers an alternative mounting option for bone transport. We found this technique particularly useful in the treatment of bone loss secondary to infection, where previous treatment and prolonged immobilisation had led to osteopenia.
